# Plasma levels of free fatty acid differ in patients with left ventricular preserved, mid-range, and reduced ejection fraction

**DOI:** 10.1186/s12872-018-0850-0

**Published:** 2018-05-29

**Authors:** Ning Zhu, Wenbing Jiang, Yi Wang, Youyang Wu, Hao Chen, Xuyong Zhao

**Affiliations:** Department of Cardiology, The Third Clinical College of Wenzhou Medical University, Wenzhou People’s Hospital, No. 57 Canghou Street, Wenzhou, 325000 Zhejiang Province People’s Republic of China

**Keywords:** Heart failure, Preserved ejection fraction, Mid-range ejection fraction, Reduced ejection fraction, Free fatty acid

## Abstract

**Background:**

Free fatty acids (FFAs) predicted the risk of heart failure (HF) and were elevated in HF with very low left ventricular ejection fraction (LVEF) compared to healthy subjects. The aim of this study was to investigate whether total levels of FFA in plasma differed in patients with HF with preserved (HFpEF), mid-range (HFmrEF), and reduced ejection fraction (HFrEF) and the association with the three categories.

**Methods:**

One hundred thirty-nine patients with HFpEF, HFmrEF and HFrEF were investigated in this study. Plasma FFA levels were measured using commercially available assay kits, and LVEF was calculated by echocardiography with the Simpson biplane method. Dyspnea ranked by New York Heart Association (NYHA) was also identified.

**Results:**

FFA concentrations were higher in HFrEF than in HFmrEF and HFpEF, respectively (689 ± 321.5 μmol/L vs. 537.9 ± 221.6 μmol/L, *p* = 0.036; 689 ± 321.5 μmol/L vs. 527.5 ± 185.5 μmol/L, *p* = 0.008). No significant differences in FFA levels were found between HFmrEF and HFpEF (537.9 ± 221.6 μmol/L vs. 527.5 ± 185.5 μmol/L, *p* = 0.619). In addition, we found a negative correlation between FFA levels and LVEF (regression coefficient: − 0.229, *p* = 0.004) and a positive correlation between FFAs and NYHA class (regression coefficient: 0.214, *p* = 0.014) after adjustment for clinical characteristic, medical history and therapies. ROC analysis revealed that FFAs predicted HFrEF across the three categories (AUC: 0.644, *p* = 0.005) and the optimal cut-off level to predict HFrEF was FFA levels above 575 μmol/L.

**Conclusions:**

FFA levels differed across the three categories, which suggests that energy metabolism differs between HFpEF, HFmrEF and HFrEF.

## Background

During the past 20 years, considerable progress in the treatment has improved the survival of patients with heart failure (HF). However HF remains a leading cause of morbidity and mortality throughout the world [1].

Heart failure is a clinical syndrome characterized by typical symptoms and signs resulting from Left ventricular ejection fraction (LVEF), which has an essential role in phenotyping and guiding the therapy [2]. Patients with EF < 40% are defined as reduced EF (HFrEF), and therapies have been shown to reduce both morbidity and mortality [2, 3]. Patients with EF ≥50% are generally considered as preserved EF (HFpEF), and therapies mainly directing at symptoms, comorbidities and risk factors, failed to confer a survival benefit [4, 5]. A ‘grey zone’ of EF 40–49% was formally termed as heart failure with mid-range ejection fraction (HFmrEF) in the recent European Society of Cardiology (ESC) HF guidelines. However, direct evidence on this group remains lacking and whether HFmrEF patients are characterized by diverse demographic, or clinical features, different co-morbidities and distinct response to therapies should be compared to HFpEF or HFrEF [6]. Identifying HFmrEF as a separate group will stimulate research into the underlying characteristics, pathophysiology and treatment of this group of patients, and contribute to the better understanding of HF.

Under physiological conditions, Free fatty acids (FFAs) releasing from adipose tissue are the major energy sources of the heart, and fatty acids (FAs) are active components of biological membranes [7]. Although FFAs yield the highest ATP, β-oxidation of FFAs uses more oxygen than glycolysis metabolism. Hence, FFAs are less energy efficient and increase the burden of the myocardium in the patients with HF. Furthermore, numerous evidences suggest that blocking fatty acid oxidation and increasing glucose oxidation can improve cardiac contractile function, leading to improve prognosis in patients with HF [8, 9]. In general, circulating FFAs may be a crucial regulator of myocardial substrate metabolism in HF.

The composition of FFAs could also influence myocardial function. It is well known that elevated circulating FFA levels could cause chronic inflammation, insulin resistance, and cardiovascular disease [10, 11]. These processes could occur in many tissues such as the heart, liver, skeletal muscle and pancreas. Previous studies showed that patients with HF had higher plasma FFAs than healthy controls [12]. Moreover, FFAs were independently associated with incident HF in older adults [13]. However, FFA levels in patients with HFpEF, HFmrEF and HFrEF and the association of FFAs with the three categories remains unknown.

Thus, the main aim of this observatory study was to investigate whether total levels of FFA in plasma differed across the three categories and the association of FFA plasma levels with the extent of heart failure with the three categories.

## Methods

### Study population

A total of 139 men and women were enrolled from Wenzhou People’s Hospital. All these patients were diagnosed with chronic HF according to contemporary guidelines. In addition, all the patients were symptomatic and were treated according to contemporary clinical guidelines. Patients were excluded from the study if they had any recent acute coronary syndrome, stroke, immune system disorders, severe valvular disease, or any other concomitant terminal disease. Upon entering the study, the set of baseline variables including previous clinical history, treatments, the gender, height, and weight of all the patients were collected. LVEF were calculated by echocardiography with the Simpson biplane method. Based on the LVEF measured at time of inclusion, patients were categorized as HFpEF as LVEF ≥50%, HFmrEF as LVEF 40–49%, and HFrEF as LVEF < 40%.

### Biochemical measurements

Following venous blood sample collection, Blood was drawn into chilled glass tubes containing EDTA, placed on ice and centrifuged at 3000 rpm for 10 min. The separated serum specimens were immediately frozen and stored at − 80 °C until the time of the assay. The FFA serum levels were measured on biochemical instrument (Beckman Coulter, USA) by a commercially available FFA kit (Reebio, Ningbo, China). All of assays were conducted according to manufacturer’s guidelines.

### Ethical considerations

The study complied with the Declaration of Helsinki and was approved by the Wenzhou People’s Hospital ethics committee, and all patients gave written informed consent.

### Statistical analysis

All the data are presented as mean ± SD. As the data included continuous variables and classification variables, parameter and non-parameter methods were both used. Non-parametric tests were also used in case of non-normally distributed data. Comparisons were made by Pearson chi-square for proportions and Mann–Whitney test and Kruskal–Wallis test for continuous variables. The correlation between plasma FFAs and HF risk factors (hypertension, diabetes, weight, BMI, CHD), LVEF and New York Heart Association (NYHA) class was assessed by Spearman rank correlation test. Multiple regression analysis with input selection method was used to adjust gender, smoking, hypertension, diabetes, coronary heart disease, prior revascularization, prior myocardial infarction, dilated myocardiopathy, atrial fibrillation, diuretics, aldosteroneantagonists, beta-blockers, ACEI/ARB, digitoxin, statins and warfarin. The receiver operating characteristic (ROC) curves for associations between FFAs, as well as NT-proBNP and HFpEF, HFmrEF, and HFrEF compared. All *P*<0.05 were considered significant.

## Results

A total of 139 patients was classified as HFpEF (*n* = 51, 36.6%), HFmrEF (*n* = 39, 28.1%), and HFrEF (*n* = 49, 35.3%). Table 1 shows the baseline characteristics of the study population, and most of clinical characteristics differ across the three categories. HFmrEF was close to HFpEF and HFrEF in terms of age, however HFmrEF were more often male than HFpEF and less often male than HFrEF. In addition, the prevalence of hypertension, diabetes and dilated myocardiopathy were higher in HFmrEF than in HFpEF, but lower than in HFrEF.

**Table 1:** Characteristics according to categories of left ventricular ejection fraction (LVEF)

	HFpEF	HFmrEF	HFrEF	Overall	HFpEF vs. HFmrEF	HFmrEF vs. HFrEF	HFpEF vs. HFrEF
	(*n* = 51 36.6%)	(*n* = 39 28.1%)	(*n* = 49 35.3%)	*P*	*P*	*P*	*P*
Characteristic							
Age, years	79.3 ± 8.9	77.2 ± 10.2	78.0 ± 11.8	0.553	0.297	0.626	0.501
Male gender	30(58.8%)	26(66.6%)	42(85.7%)	<0.001	<0.05	<0.001	<0.001
Current smoking	15(29.4%)	12(30.7%)	15(30.6%)	<0.001	<0.001	<0.001	<0.001
Hypertension	33(64.7%)	28(71.7%)	40(81.6%)	<0.001	0.001	<0.001	<0.001
Diabetes	12(23.5%)	12(30.7%)	16(32.6%)	<0.001	<0.001	0.001	<0.001
NYHA III/IV	37(72.5%)	30(76.9%)	44(89.7%)	<0.001	<0.001	<0.001	<0.001
LVEF(%)	60.1 ± 6.6	44.5 ± 2.9	32.6 ± 4.1	<0.001	<0.001	<0.001	<0.001
Weight(kg)	59.4 ± 10.8	60.9 ± 10.2	60.0 ± 10.9	0.735	0.143	0.644	0.799
BMI	22.7 ± 4.0	22.9 ± 3.0	22.0 ± 3.2	0.791	0.654	0.775	0.526
Nt-proBNP	5825.8 ± 6484.2	9664 ± 10,189.1	11,248.1 ± 10,875.1	<0.05	<0.05	0.408	<0.05
Other medical history							
Coronary heart disease	41((80.3%)	29(74.3%)	39(79.5%)	<0.001	<0.001	<0.001	<0.001
Prior revascularization	10(19.6%)	7(17.9%)	17(34.6%)	<0.001	<0.001	<0.001	<0.001
Prior myocardial infarction	6(11.7%)	9(23%)	8(16.3)	<0.001	<0.001	<0.001	<0.001
Dilated myocardiopathy	0(0%)	5(12.8%)	12(24.4%)	<0.001	<0.001	<0.001	<0.001
Atrial fibrillation	29(56.8%)	17(43.5%)	16(32.6%)	0.203	0.833	<0.05	0.317
Therapies							
Diuretics	39(76.4%)	38(97.4%)	42(85.7%)	<0.001	<0.001	<0.001	<0.001
Aldosteroneantagonists	38(77.5%)	26(66.6%)	34(69.3%)	<0.001	<0.001	<0.05	<0.001
Beta-blockers	35(68.6%)	21(53.8%)	24(48.9%)	0.075	<0.05	0.831	0.072
ACEI/ARB	32(61.5%)	21(53.8%)	32(65.3%)	<0.05	0.092	0.055	<0.05
Digitoxin	8(15.6%)	10(25.6%)	22(44.8%)	<0.001	<0.001	<0.05	<0.001
Statins	34(66.6%)	28(71.7%)	33(67.3%)	<0.001	<0.001	<0.001	0.001
Warfarin	15(29.4%)	6(15.3%)	6(12.2%)	<0.001	<0.001	<0.001	<0.001

Data are presented as mean ± SD, or number or percentage of subjects

*NYHA* New York Heart Association, *LVEF* left ventricular ejection fraction, *BMI* body Mass Index, *Nt-proBNP* N-terminal pro-B-type natriuretic peptide, *ACE* angiotensin-converting enzyme, *ARB* angiotensin II receptor blocker, *p* significance level

There was more smoking, prior myocardial infarction in HFmrEF than in HFpEF and HFrEF while there was less coronary heart disease (CHD) and prior revascularization in HFmrEF than in HFpEF and HFrEF. There were no differences in weight and BMI between HFpEF, HFmrEF, and HFrEF, however HFmrEF had higher weight than HFpEF. HFmrEF had more atrial fibrillation than HFrEF, but similar N-Terminal pro-brain natriuretic peptide (NT-proBNP) levels than HFrEF. NYHA III/IV was intermediate in HFmrEF.

Regarding therapies, there were highest use of diuretics and statins, intermediate use of digitoxin and warfarin, and lowest use of aldosteroneantagonist in HFmrEF. HFmrEF had similar rate of angiotensin-converting enzyme (ACE)-inhibitors and angiotensin receptor blockers (ARBs) and lower rate of Beta-blocker than HFpEF. It was also found that The HFmrEF group had intermediate rate of digitoxin.

**Fig. 1:**
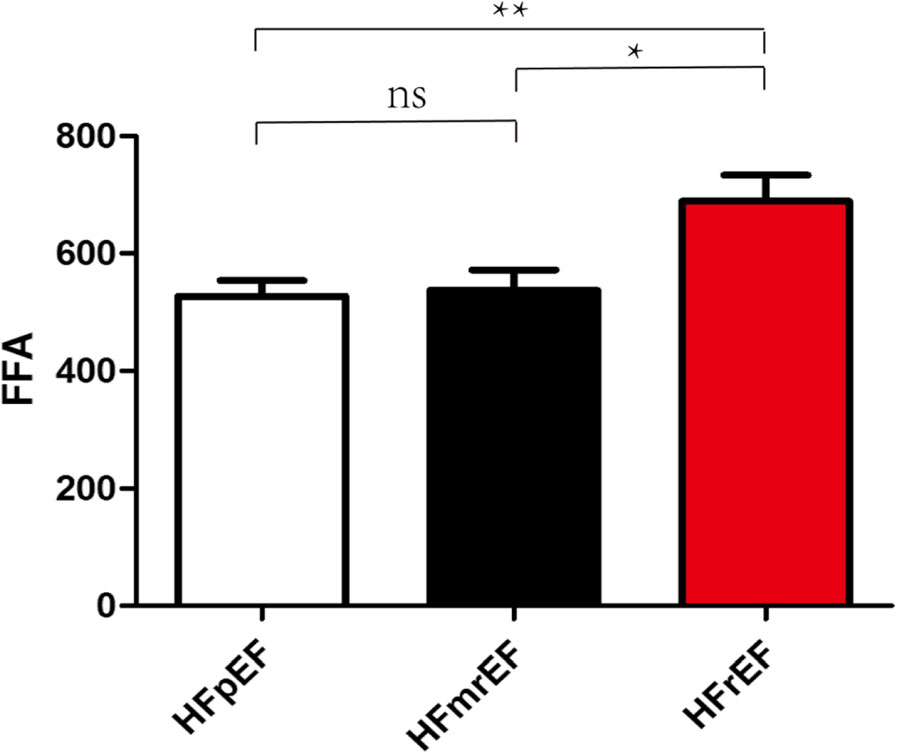
Plasma FFA levels in patients with HFpEF, HFmrEF and HFrEF. *P<0.05, **P<0.01

**Fig. 2:**
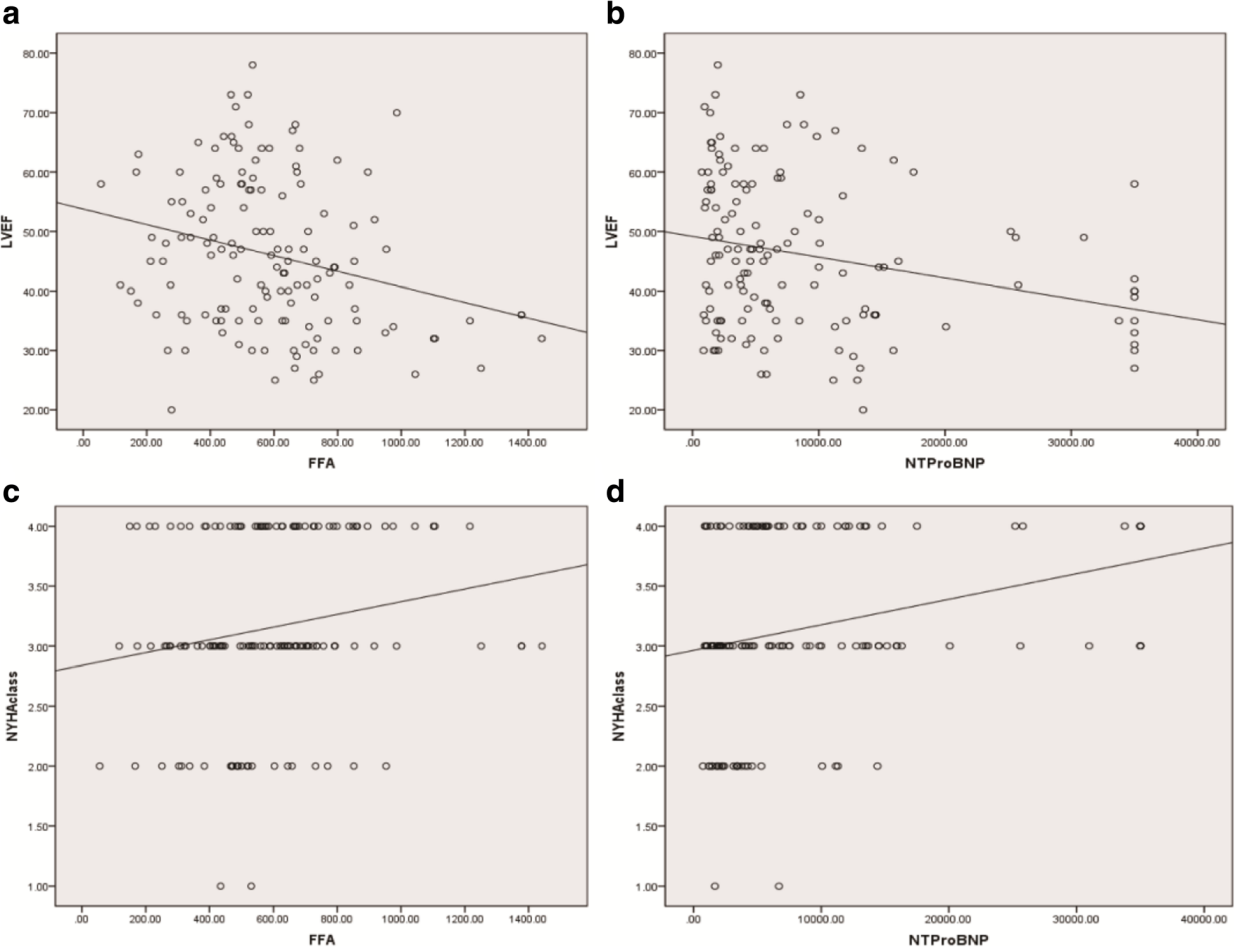
Association of FFA levels and NT-proBNP with left ventricular ejection fraction (**a**, **b**) and NYHA class (**c**, **d**)

**Fig. 3:**
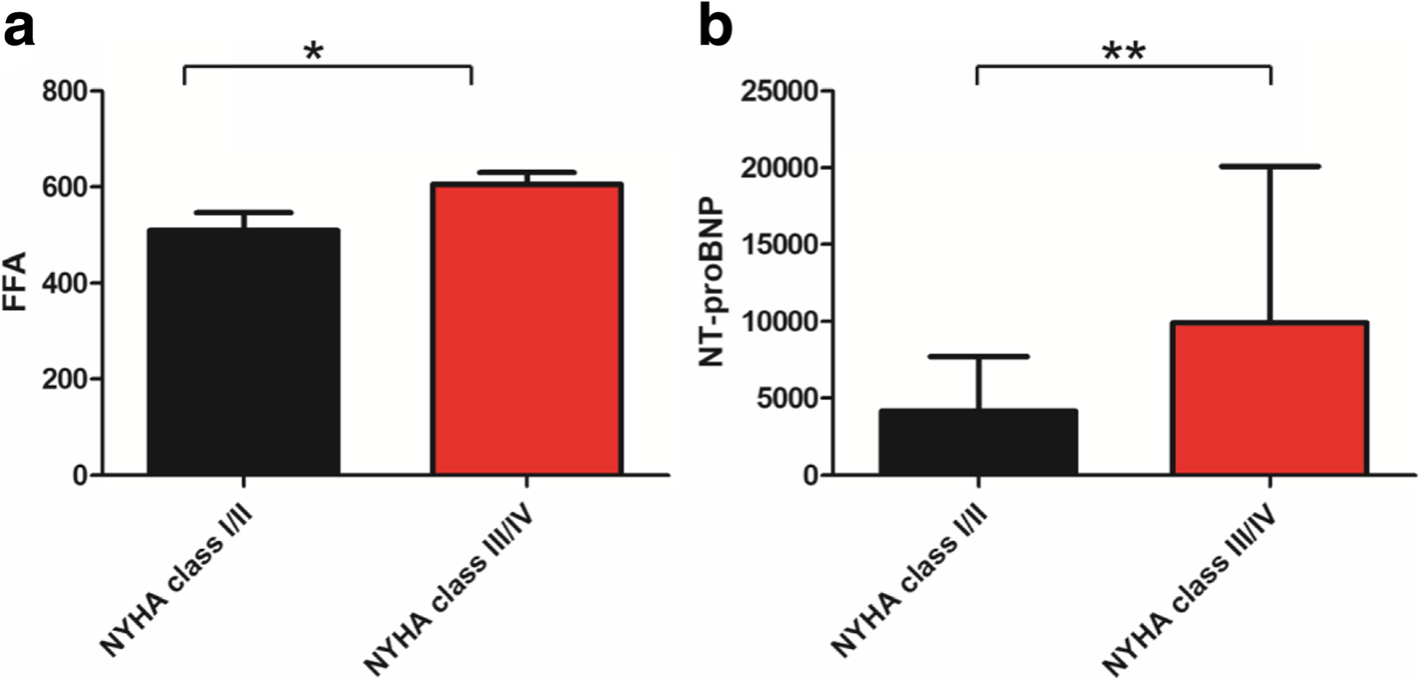
Plasma FFA levels (**a**) and NT-proBNP levels (**b**) in patients with NYHA class I/II and NYHA class III/IV. *P<0.05, **P<0.01

**Fig. 4:**
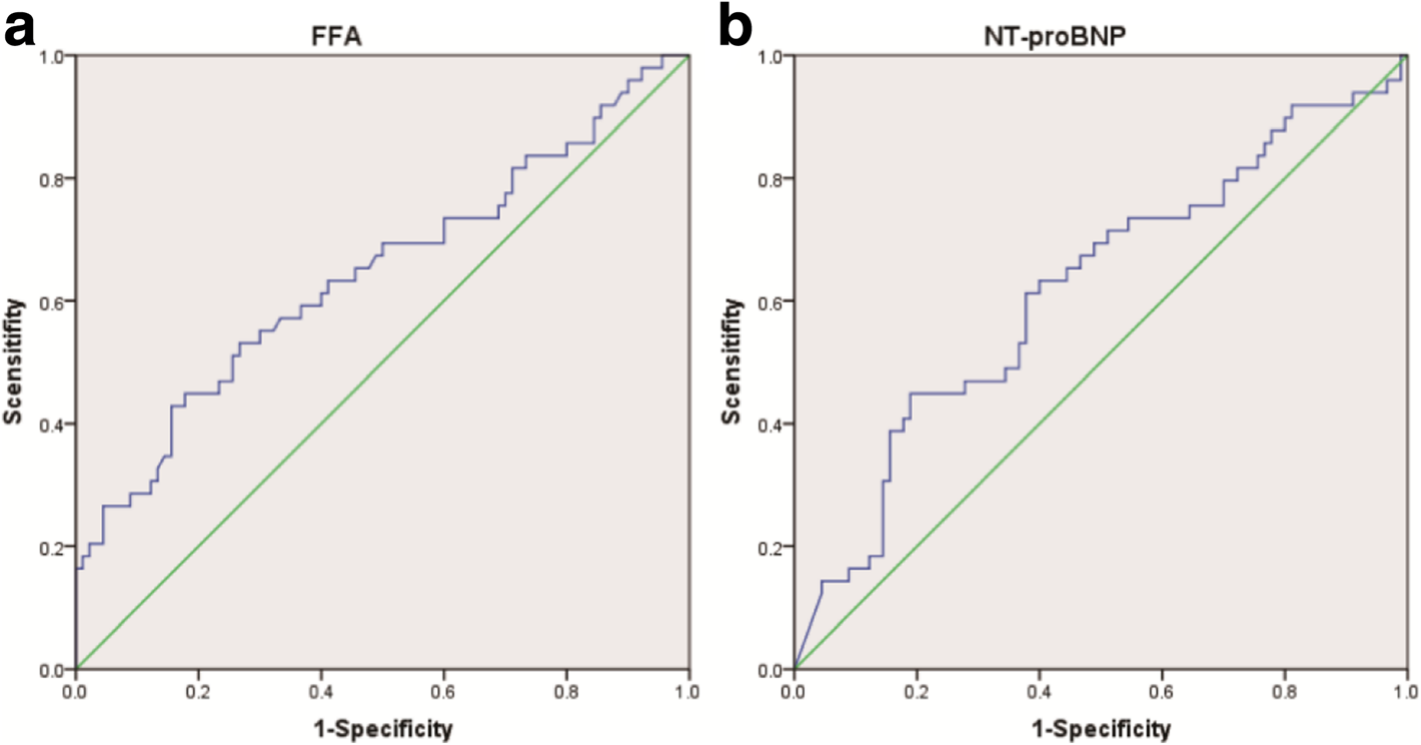
ROC analysis of FFA (**a**) and NT-proBNP (**b**) in prediction of HFrEF

FFA concentrations were higher in HFrEF than in HFmrEF and HFpEF, respectively (689 ± 321.5 μmol/L vs. 537.9 ± 221.6 μmol/L, *p* = 0.036; 689 ± 321.5 μmol/L vs. 527.5 ± 185.5 μmol/L, *p* = 0.008, Fig. 1). No significant differences in levels of FFA were found between HFmrEF and HFpEF (537.9 ± 221.6 μmol/L vs. 527.5 ± 185.5 μmol/L, *p* = 0.619, Fig. 1). Our data showed FFAs were associated with hypertension (regression coefficient: 0.156, *p* = 0.033, Table 2), but there was no significant correlation between plasma FFA levels and diabetes, weight, BMI, CHD (regression coefficient: 0.092, *p* = 0.28; regression coefficient: 0.092, *p* = 0.28; regression coefficient: 0.151, *p* = 0.076; regression coefficient: 0.00004, *p* = 0.713 Table 2). There was still no significant correlation between plasma FFA levels and weight, BMI and CHD even after adjusting for gender, smoking, hypertension, diabetes, coronary heart disease, prior revascularization, prior myocardial infarction, dilated myocardiopathy, atrial fibrillation, diuretics, aldosteroneantagonists, beta-blockers, ACEI/ARB, digitoxin, statins and warfarin (regression coefficient: 0.114, *p* = 0.183; regression coefficient: 0.214, *p* = 0.117; regression coefficient: 0.097, *p* = 0.459, Table 3). In contrast, we found a negative correlation between FFA levels and LVEF (regression coefficient: − 0.267, *p* = 0.001, Fig. 2a). A negative correlation was also found between NT-proBNP and LVEF (regression coefficient: − 0.264, *p* = 0.002, Fig. 2b). Moreover, there was a positive correlation between FFAs as well as NT-proBNP and NYHA class (regression coefficient: 0.202, *p* = 0.017, Fig. 2c; regression coefficient: 0.302, *p* < 0.001, Fig. 2d). FFA levels were higher in patients with NYHA class III and IV than in patients with NYHA class I and II (605.3 ± 264 μmol/L vs. 509.2 ± 189 μmol/L, *p* = 0.034, Fig. 3a). Meanwhile, patients with NYHA class III and IV have also higher NT-proBNP (9933.5 ± 10,156 μmol/L vs. 4171 ± 3545 μmol/L, *p* = 0.0044, Fig. 3b) Multiple regression analysis showed FFAs still significantly correlated with LVEF and NYHA class after adjustment for gender, smoking, hypertension, diabetes, coronary heart disease, prior revascularization, prior myocardial infarction, dilated myocardiopathy, atrial fibrillation, diuretics, aldosteroneantagonists, beta-blockers, ACEI/ARB, digitoxin, statins and warfarin (regression coefficient: − 0.229, *p* = 0.004; regression coefficient: 0.214, *p* = 0.014, Table 3). Similar results were found in the correlation between NT-proBNP and LVEF and NYHA class (regression coefficient: − 0.101, *p* = 0.025; regression coefficient: 0.234, *p* = 0.012). ROC analysis revealed FFA predicted HFrEF across the three categories (AUC: 0.644, *p* = 0.005, Fig. 4a) and the optimal cut-off level to predict HFrEF were FFA levels above 575 μmol/L. ROC analysis showed NT-proBNP also predicted HFrEF across the three categories (AUC: 0.619, *p* = 0.021, Fig. 4b).

**Table 2:** Association of FFA levels with diabetes, hypertension, weight, BMI and CHD

	Regression coefficient	*P*
Diabetes	0.092	0.28
Hypertension	0.156	0.033
Weight	0.092	0.28
BMI	0.151	0.076
CHD	0.00004	0.713

*BMI* body Mass Index, *CHD* coronary heart disease *p* significance level

**Table 3:** Multiple regression analysis

	Regression coefficient	*P*
Weight	0.114	0.183
BMI	0.142	0.117
CHD	0.097	0.459
LVEF	−0.229	0.004
NYHA class	0.214	0.014

*BMI* body Mass Index. *CHD* coronary heart disease, *LVEF* Left ventricular ejection fraction, *NYHA* New York Heart Association, *p* significance level

## Discussion

This is a pilot study to compare FFAs in HFpEF, HFmrEF, and HFrEF based on the newly defined HF types in the 2016 ESC guideline. Despite a clinical profile similar to those with HFpEF or HFrEF, patients with HFmrEF have many different characteristics. In line with previous researches [14–16], our results suggested that patients with HFmrEF constituted a specific HF phenotype. In addition, we found that HFrEF had higher plasm FFA levels than HFpEF and HFmrEF. However, plasma FFA levels in HFmrEF were similar to that in HFpEF. Plasm FFA levels were negatively associated with LVEF and positively associated with NYHA class, which were similar to NT-proBNP. ROC curve showed that FFAs and NT-proBNP predicted HFrEF across the three categories.

There were limited studies specifically comparing HFpEF, HFmrEF and HFrEF, especially in Chinese population. Our study showed that many clinical characteristics such as gender, hypertension, dilated myocardiopathy and atrial fibrillation were on a continuum between HFpEF and HFrEF but weight and BMI were similar in the three categories. In addition, HFmrEF had higher rates of coronary artery disease and revascularization.

To the best of our knowledge, this is the first study to examine whether the association of plasma FFA concentration with HFpEF, HFmrEF, and HFrEF. The associations between FFAs and risk factors including hypertension, atrial fibrillation, diabetes mellitus, and CHD for HF had rarely been reported. Free fatty acid elevation has been identified as a highly significant risk factor for hypertension [17]. Furthermore, it has been demonstrated that elevated FFAs are positively associated with systolic blood pressure in men and diastolic blood pressure in women [18]. However, the prevalence of diabetes in parents was similar in the highest and lowest quartiles as well as in both diabetic and nondiabetic parents. Previous study also showed that FFA levels modulated microvascular function and subsequently resulted in obesity-associated insulin resistance and hypertension [19]. Moreover, a positive association between plasma FFAs and incident diabetes was observed during the first 5 years of follow-up [20]. Although several studies reported that plasma FFAs were associated with CHD [18, 21]. FFA concentrations were not associated with CHD death in the Paris Prospective Study [22]. In this study, we found there was a positive association of FFAs and hypertension, but not diabetes and CHD. Therefore, the association of FFAs with diabetes and CHD remains controversial.

Plasma FFA levels were increased in the obese state and could be normalized by reducing body mass [23, 24], which was probably due to an increased adipose tissue insulin resistance [25]. Our study showed that FFA levels were not associated with diabetes in HF, which suggests that insulin resistance may not cause an increase in FFAs. Increased plasma FFAs are also associated with an increased cardiac fatty acid uptake. Interestingly, our study also showed that FFAs were not associated with weight and BMI, and BMI showed values within the normal range in HFpEF, HFmrEF, and HFrEF. Therefore, increased FFAs in HF may be due to an increase in energy demand and lead to impaired left ventricular function [26]. The structural and metabolic alterations of myocardial cells consequent to HF are due to reduction in glucose utilization and increase in fatty acid utilization. It has been also demonstrated that Inflammation and oxidative stress induced by FFAs are involved in impaired heart function [27–29].

In accordance with NT-proBNP, our data showed FFA levels were positively associated with NYHA class and negatively associated with LVEF in patients with HFpEF, HFmrEF, and HFrEF. Moreover, patients with NYHA class III and IV had higher FFA levels than in patients with NYHA class I and II, which was also similar to NT-proBNP. These findings suggest FFA levels are associated with heart function. Although acipimox reduced circulating FFAs by − 69% and heart function in Type 2 diabetes (T2DM) patients. [30]. However, 8 T2DM patients had very high LVEF (78 ± 8). And in the setting of HF, patients have different internal environment, pathophysiological state and impaired LVEF. Furthermore, the sample size of this study was very small. Therefore, according to the present study, in the setting of HF, high levels of FFA lead to impaired LVEF. Further, trimetazidine improved left ventricular function in elderly patients with coronary artery disease and low LVEF [31]. As was showed in our study, FFAs were much higher in HFrEF than in HFpEF and HFmrEF but HFpEF shared similar FFAs with HFmrEF. In addition, ROC analysis showed that high FFA levels predicted HFrEF. Interestingly, ROC analysis also showed NT-proBNP predicted HFrEF across the three categories, which suggest HFrEF is great distinct from HFpEF and HFmrEF. Our findings indicated that FFAs were more likely to affect systolic heart function and patients with HFrEF. By contrast, HFmrEF may be similar to HFpEF in term of energy metabolism. Although elevated FFAs could also induce inflammation and oxidative stress and there was an association between FFAs and mortality [12], patients with HF still could gain benefit from inhibition of fatty acid oxidation [32, 33]. And our study indicates that patients with HFrEF may gain much more benefit. Directly lowering FFAs could be another effective treatment for HFrEF. Hence, plasma FFAs could help identify HFrEF across three categories and the state of energy metabolism, contributing to instruct drug therapy. Further study should be conducted to identify whether FFA levels have these effects on patients with HFrEF.

The present study has a few limitations. We could not exclude an excess intake of FAs could have influenced our data. Nonfasting samples were used in the study. Our study did not show whether mortality differed in three categories and was associated with FFA levels by follow-up. Further studies are required to identify whether inhibition of fatty acid oxidation or directly lowering FFAs are efficient treatment for three categories, especially HFrEF.

## Conclusions

HFmrEF exhibited a new HF type. We found that there is a positive association of FFAs and hypertension, but not diabetes, weight, BMI in HF. Further, FFA levels were positively associated with NYHA class and negatively associated with LVEF in patients with HFpEF, HFmrEF, and HFrEF. Our data also suggest that plasma FFA levels reflect systolic heart function and could predict HFrEF.

## Abbreviations

FFA: free fatty acid
HFmrEF: Heart failure with mid-range ejection fraction
HFpEF: Heart failure with preserved ejection fraction
HFrEF: Heart failure with reduced ejection fraction
LVEF: left ventricular ejection fraction
NYHA: New York heart association

## References

[1] Najafi F, Jamrozik K, Dobson AJ (2009). Understanding the ‘epidemic of heart failure’: a systematic review of trends in determinants of heart failure. Eur J Heart Fail.

[2] Ponikowski P, Voors AA, Anker SD, Bueno H, Cleland JG, Coats AJ, Falk V, González-Juanatey JR, Harjola VP, Jankowska EA, Jessup M, Linde C, Nihoyannopoulos P, Parissis JT, Pieske B, Riley JP, Rosano GM, Ruilope LM, Ruschitzka F, Rutten FH, van der Meer P; Authors/Task Force Members (2016) ESC Guidelines for the diagnosis and treatment of acute and chronic heart failure: The Task Force for the diagnosis and treatment of acute and chronic heart failure of the European Society of Cardiology (ESC). Developed with the special contribution of the Heart Failure Association (HFA) of the ESC. 2016 ESC guidelines for the diagnosis and treatment of acute and chronic heart failure: the task force for the diagnosis and treatment of acute and chronic heart failure of the European Society of Cardiology (ESC). Developed with the special contribution of the heart failure association (HFA) of the ESC. Eur Heart J 37(27):2129–2200. 10.1093/eurheartj/ehw128 27206819

[3] Yancy CW, Jessup M, Bozkurt B, Butler J, Casey DE, Drazner MH, Fonarow GC, Geraci SA, Horwich T, Januzzi JL, Johnson MR, Kasper EK, Levy WC, Masoudi FA, McBride PE, McMurray JJ, Mitchell JE, Peterson PN, Riegel B, Sam F, Stevenson LW, Tang WH, Tsai EJ, Wilkoff BL, American College of Cardiology Foundation; American Heart Association Task Force on Practice Guidelines (2013). 2013 ACCF/AHA guideline for the management of heart failure. A report of the American College of Cardiology Foundation/American Heart Association task force on practice guidelines. J Am Coll Cardiol.

[4] Senni M, Paulus WJ, Gavazzi A, Fraser AG, Díez J, Solomon SD, Smiseth OA, Guazzi M, Lam CS, Maggioni AP, Tschöpe C, Metra M, Hummel SL, Edelmann F, Ambrosio G, Stewart Coats AJ, Filippatos GS, Gheorghiade M, Anker SD, Levy D, Pfeffer MA, Stough WG, Pieske BM (2014). New strategies for heart failure with preserved ejection fraction: the importance of targeted therapies for heart failure phenotypes. Eur Heart J.

[5] Ohlmeier C, Mikolajczyk R, Frick J, Prütz F, Haverkamp W, Garbe E (2015). Incidence, prevalence and 1-year all-cause mortality of heart failure in Germany: a study based on electronic healthcare data of more than six million persons. Clin Res Cardiol.

[6] Butler J, Fonarow GC, Zile MR, Lam CS, Roessig L, Schelbert EB, Shah SJ, Ahmed A, Bonow RO, Cleland JG, Cody RJ, Chioncel O, Collins SP, Dunnmon P, Filippatos G, Lefkowitz MP, Marti CN, JJ MM, Misselwitz F, Nodari S, O'Connor C, Pfeffer MA, Pieske B, Pitt B, Rosano G, Sabbah HN, Senni M, Solomon SD, Stockbridge N, Teerlink JR, Georgiopoulou VV, Gheorghiade M (2014). Developing therapies for heart failure with preserved ejection fraction: current state and future directions. JACC Heart Fail.

[7] Stanley WC, Recchia FA, Lopaschuk GD (2005). Myocardial substrate metabolism in the normal and failing heart. Physiol Rev.

[8] Di Napoli P, Barsotti A (2009). Prognostic relevance of metabolic approach in patients with heart failure. Curr Pharm Des.

[9] Lopaschuk GD (2017). Metabolic modulators in heart disease – past, present and future. Can J Cardiol.

[10] Current Opinion in Endocrinology, Diabetes & Obesity. Editorial overview (2010) Curr Opin Endocrinol Diabetes Obes 17(3):187. 10.1097/MED.0b013e3283394ef4 20404725

[11] Zeng C, Zhong P, Zhao Y, Kanchana K, Zhang Y, Khan ZA, Chakrabarti S, Wu L, Wang J, Liang G (2015). Curcumin protects hearts from FFA-induced injury by activating Nrf2 and inactivating NF-κB both in vitro and in vivo. J Mol Cell Cardiol.

[12] Øie E, Ueland T, Dahl CP, Bohov P, Berge C, Yndestad A, Gullestad L, Aukrust P, Berge RK (2011). Fatty acid composition in chronic heart failure: low circulating levels of eicosatetraenoic acid and high levels of vaccenic acid are associated with disease severity and mortality. J Intern Med.

[13] Djoussé L, Benkeser D, Arnold A, Kizer JR, Zieman SJ, Lemaitre RN, Tracy RP, Gottdiener JS, Mozaffarian D, Siscovick DS, Mukamal KJ, Ix JH (2013). Plasma free fatty acids and risk of heart failure: the cardiovascular health study. Circ Heart Fail.

[14] Löfman I, Szummer K, Dahlström U, Jernberg T, Lund LH. Associations with and prognostic impact of chronic kidney disease in heart failure with preserved, mid-range, and reduced ejection fraction. Eur J Heart Fail. 2017; 10.1002/ejhf.821. 10.1002/ejhf.821 28371075

[15] Pascual-Figal DA, Ferrero-Gregori A, Gomez-Otero I, Vazquez R, Delgado-Jimenez J, Alvarez-Garcia J, Gimeno-Blanes JR, Worner-Diz F, Bardají A, Alonso-Pulpon L, Gonzalez-Juanatey JR, Cinca J, MUSIC and REDINSCOR I research groups (2017). Mid-range left ventricular ejection fraction: clinical profile and cause of death in ambulatory patients with chronic heart failure. Int J Cardiol.

[16] Farmakis D, Simitsis P, Bistola V, Triposkiadis F, Ikonomidis I, Katsanos S, Bakosis G, Hatziagelaki E, Lekakis J, Mebazaa A, Parissis J (2017). Acute heart failure with mid-range left ventricular ejection fraction: clinical profile, in-hospital management, and short-term outcome. Clin Res Cardiol.

[17] Fagot-Campagna A, Balkau B, Simon D, Warnet JM, Claude JR, Ducimetière P, Eschwège E (1998). High free fatty acid concentration: an independent risk factor for hypertension in the Paris prospective study. Int J Epidemiol.

[18] Carlsson M, Wessman Y, Almgren P, Groop L (2000). High levels of nonesterified fatty acids are associated with increased familial risk of cardiovascular disease. Arterioscler Thromb Vasc Biol.

[19] de Jongh RT, Serné EH, Ijzerman RG, de Vries G, Stehouwer CD (2004). Free fatty acid levels modulate microvascular function: relevance for obesity-associated insulin resistance, hypertension, and microangiopathy. Diabetes.

[20] Djoussé L, Khawaja O, Bartz TM, Biggs ML, Ix JH, Zieman SJ, Kizer JR, Tracy RP, Siscovick DS, Mukamal KJ (2012). Plasma fatty acid-binding protein 4, nonesterified fatty acids, and incident diabetes in older adults. Diabetes Care.

[21] Westphal S, Gekeler GH, Dierkes J, Luley C (2002). A free fatty acid tolerance test identifies patients with coronary artery disease among individuals with a low conventional coronary risk profile. Heart Vessel.

[22] Charles MA, Fontbonne A, Thibult N, Claude JR, Warnet JM, Rosselin G, Ducimetière P, Eschwège E (2001). High plasma nonesterified fatty acids are predictive of cancer mortality but not of coronary heart disease mortality: results from the Paris prospective study. Am J Epidemiol.

[23] Normand-Lauzière F, Frisch F, Labbé SM, Bherer P, Gagnon R, Cunnane SC, Carpentier AC (2010). Increased postprandial nonesterified fatty acid appearance and oxidation in type 2 diabetes is not fully established in offspring of diabetic subjects. PLoS One.

[24] Il'yasova D, Wang F, D'Agostino RB, Hanley A, Wagenknecht LE (2010). Prospective association between fasting NEFA and type 2 diabetes: impact of post-load glucose. Diabetologia.

[25] Lionetti L, Mollica MP, Lombardi A, Cavaliere G, Gifuni G, Barletta A (2009). From chronic overnutrition to insulin resistance: the role of fat-storing capacity and inflammation. Nutr Metab Cardiovasc Dis.

[26] Kankaanpää M, Lehto HR, Pärkkä JP, Komu M, Viljanen A, Ferrannini E, Knuuti J, Nuutila P, Parkkola R, Iozzo P (2006). Myocardial triglyceride content and Epicardial fat mass in human obesity: relationship to left ventricular function and serum free fatty acid levels. J Clin Endocrinol Metab.

[27] van de Weijer T, Schrauwen-Hinderling VB, Schrauwen P (2011). Lipotoxicity in type 2 diabetic cardiomyopathy. Cardiovasc Res.

[28] Eguchi K, Manabe I, Oishi-Tanaka Y, Ohsugi M, Kono N, Ogata F, Yagi N, Ohto U, Kimoto M, Miyake K, Tobe K, Arai H, Kadowaki T, Nagai R (2012). Saturated fatty acid and TLR signaling link β cell dysfunction and islet inflammation. Cell Metab.

[29] Wahli W, Michalik L (2012). PPARs at the crossroads of lipid signaling and inflammation. Trends Endocrinol Metab.

[30] Wolf P, Winhofer Y, Krssak M, Smajis S, Harreiter J, Kosi-Trebotic L, Fürnsinn C, Anderwald CH, Baumgartner-Parzer S, Trattnig S, Luger A, Krebs M (2016). Suppression of plasma free fatty acids reduces myocardial lipid content and systolic function in type 2 diabetes. Nutr Metab Cardiovasc Dis.

[31] Vitale C, Wajngaten M, Sposato B, Gebara O, Rossini P, Fini M, Volterrani M, Rosano GM (2004). Trimetazidine improves left ventricular function and quality of life in elderly patients with coronary artery disease. Eur Heart J.

[32] Gao D, Ning N, Niu X, Hao G, Meng Z (2011). Trimetazidine: a meta-analysis of randomised controlled trials in heart failure. Heart.

[33] Zhang L, Lu Y, Jiang H, Zhang L, Sun A, Zou Y, Ge J (2012). Additional use of Trimetazidine in patients with chronic heart failure. J Am Coll Cardiol.

